# A novel prognostic model based on cellular senescence-related gene signature for bladder cancer

**DOI:** 10.3389/fonc.2022.937951

**Published:** 2022-11-23

**Authors:** Lianmin Luo, Fenghua Li, Binbin Gong, Ping Xi, Wenjie Xie

**Affiliations:** ^1^ Department of Urology, The First Affiliated Hospital of Nanchang University, Nanchang, Jiangxi, China; ^2^ Department of Obstetrics and Gynecology, The First Affiliated Hospital of Nanchang University, Nanchang, Jiangxi, China

**Keywords:** cellular senescence, bladder cancer, prognosis signature, immune infiltration, biomarkers

## Abstract

**Background:**

Cellular senescence plays crucial role in the progression of tumors. However, the expression patterns and clinical significance of cellular senescence-related genes in bladder cancer (BCa) are still not clearly clarified. This study aimed to establish a prognosis model based on senescence-related genes in BCa.

**Methods:**

The transcriptional profile data and clinical information of BCa were downloaded from TCGA and GEO databases. The least absolute shrinkage and selection operator (LASSO), univariate and multivariate Cox regression analyses were performed to develop a prognostic model in the TCGA cohort. The GSE13507 cohort were used for validation. Gene ontology (GO), Kyoto Encyclopedia of Genes and Genomes (KEGG), and single-sample gene set enrichment analysis (ssGSEA) were performed to investigate underlying mechanisms.

**Results:**

A six-gene signature (CBX7, EPHA3, STK40, TGFB1I1, SREBF1, MYC) was constructed in the TCGA databases. Patients were classified into high risk and low risk group in terms of the median risk score. Survival analysis revealed that patients in the higher risk group presented significantly worse prognosis. Receiver operating characteristic (ROC) curve analysis verified the moderate predictive power of the risk model based on the six senescence-related genes signature. Further analysis indicated that the clinicopathological features analysis were significantly different between the two risk groups. As expected, the signature presented prognostic significance in the GSE13507 cohort. Functional analysis indicated that immune-related pathways activity, immune cell infiltration and immune-related function were different between two risk groups. In addition, risk score were positively correlated with multiple immunotherapy biomarkers.

**Conclusion:**

Our study revealed that a novel model based on senescence-related genes could serve as a reliable predictor of survival for patients with BCa.

## Introduction

Bladder cancer (BCa) is one of the most life-threatening cancer worldwide, with nearly 8,3730 new cases and 1,7200 deaths in the United States in 2021 according to cancer statistics (1). BCa ranges from non–muscle-invasive bladder carcinoma (NMIBC) and muscle invasive bladder carcinoma (MIBC) according to whether the tumors invades the muscle layer of the bladder (2). Despite undergoing radical cystectomy, nearly 50% of MIBC patients still have lethal metastatic recurrence, with the 5-year overall survival rate <50% (3). Therefore, it is crucial to explore the possible therapeutic target and novel prognostic biomarkers for improving the clinical outcome of patients in BCa to guide clinical practice.

Cellular senescence is a durable cell cycle arrest wherein cells fail to proliferate but remain metabolically active (4). Cellular senescence is a very complex stress response that manipulating several physiological and pathological processes, including embryogenesis (5), wound healing (6), tissue reprogramming (7) and ageing (8). In recent years, the complex relationship between cellular senescence and cancer has incited growing interest (9, 10). In cancer, senescence works as a potent inhibitor of cell cycle to suppress the proliferation of cancer cells in mammals (11, 12). Thus, because of its tumor-suppressive effects, therapy-induced senescence, such as cytotoxic chemotherapy or radiation, also can represent a therapeutic regimen for cancer (13, 14). Paradoxically, senescence cells can evade the immune system and accumulate in tissues and secrete a variety of pro-inflammatory and growth-stimulatory molecules, commonly referred to as the senescence-associated secretory phenotype (SASP) which is now recognized as a significant driver of tumor growth, relapse, and metastasis (15, 16). Consistent with this notion, mounting evidence demonstrated that cellular senescence can promote cancer initiation, invasion, and metastasis *via* the SASP that can act in autocrine and paracrine fashion to recruit proinflammatory cells that can modify the tumour microenvironment, which in turn can modulate tumour development (17, 18). In recent years, a growing studies have investigated gene expression patterns for risk stratification of patients and constructing survival prediction models in cancer (19, 20). However, the expression patterns and the potential clinical prognosis value of senescence-related genes in BCa have not yet been systematically analyzed.

In the present study, a prognostic model was constructed based on the six senescence-related genes signature in the TCGA cohort. Then, we validated the predictive power of the model in the GEO cohort. Finally, functional enrichment analysis were performed to investigate the underlying mechanisms.

## Materials and methods

### Data and resources

The data of RNA-sequencing data (FPKM values) and corresponding clinical information of BCa were downloaded from TCGA database (https://genomecancer.ucsc.edu) as training cohort. Patients were excluded if one or more clinical characteristics were incomplete. Finally, a total of 406 patients were included from TCGA database. The GSE13507 was downloaded from the gene expression omnibus database (GEO: https://www.ncbi.nlm.nih.gov/geo/) as a validation cohort.

### Identification of senescence-related differentially expressed genes

The list of senescence-related genes were collected from the CellAge database (**Supplementary Table 1**). The “limma” R package used to identify the senescence-related differentially expressed genes (DEGs) between tumor tissues and normal tissues based on the screening criteria of false discovery rate (FDR) < 0.05 and |log_2_FC| > 1.

### Construction and validation of a prognostic senescence-related gene model

Univariate Cox regression analysis was used to screen senescence-related genes with prognostic values (*P* < 0.05). The Least Absolute Shrinkage and Selection Operator (LASSO) regression with “glmnet” R package was applied in order to avoid overfitting. Subsequently, the candidate genes selected into multivariate Cox regression analysis in order to determine final prognostic senescence-related genes. Finally, the risk score of each samples was calculated based on the amount of senescence-related genes expression and corresponding coefficients. The calculation formula was as follows: risk score =β_mRNA1_×Expression_mRNA1_+β_mRNA2_×Expression_mRNA2_+β_mRNA3_×Expression_mRNA3_+…+ β_mRNAn_×Expression_mRNAn_.

The patients were classified into high risk and low risk group based on the median value of the risk score. Kaplan-Meier analysis was performed to compare the overall survival between high risk and low risk group.The receiver operating characteristic (ROC) curve analysis was used to evaluate the predictive accuracy of the senescence-related genes signature.

Parameters including age, gender, grade, TNM staging system, stage, and risk score were analyzed by univariate analysis. The candidate parameters p < 0.05 in univariate analysis selected into multivariate analysis in order to determine independent prognostic factors.

### Comprehensive analysis of the prognostic model

Parameters including age, gender, grade, TNM staging system, stage, and risk score were analyzed by univariate analysis. The candidate parameters *P* < 0.05 in univariate analysis selected into multivariate analysis in order to determine independent prognostic factors.

To further investigate the correlations between the two risk groups, clinical variables and senescence-related genes, we compared the differences in age, gender, grade, TNM staging system, stage, and senescence-related genes between the high risk and low risk group. The results were displayed as a heatmap.

### Functional and pathway enrichment analysis

The “limma” R package was applied to analyze the DEGs between the high risk and low risk group based on the screening criteria of false discovery rate (FDR) < 0.05 and |log_2_FC| > 1. Gene Ontology (GO) analysis and Kyoto Encyclopedia of Genes and Genomes (KEGG) pathway analysis were conducted to identify the biological functions and the signaling pathways that were associated with the DEGs.

### Depicting immune infiltration characteristics

Single-sample gene set enrichment analysis (ssGSEA) was used to estimate the infiltrating scores of the 16 immune cells subsets and 13 immune-related pathways between the high risk and low risk groups.

### Evaluation of inflammatory response–related genes characteristics and EMT phenotype

Inflammatory response–related genes were downloaded from the Molecular Signatures Database (MSigDB) (**Supplementary Table 1**) and compared between the high risk and low risk groups. The epithelial-to-mesenchymal transition (EMT) phenotype was evaluated based on the expression of EMT marker genes including SNAI1, SNAI2, ZEB1, ZEB2, TWIST1, Vimentin, Fibronectin, N-cadherin, and E-cadherin.

### RNA extraction and quantitative real-time PCR analysis

Total RNA was extracted from 5 paired human BCa tissues and adjacent non-tumorous tissues using TRIzol Reagent (Invitrogen). The reverse transcription was conducted with PrimeScript™ RT reagent Kit (TaKaRa). Q-PCR was done with SYBR green Premix Ex Taq II (Takara). GAPDH was selected as an internal control. The sequence of primers were listed in **Supplementary Table 2**.

### Statistical analysis

The statistical analyses were performed using the R software (Version R-4.1.2) and GraphPad Prism 8.0. Student’s t-test was applied to compare the difference between binary groups with continuous variables. *P* < 0.05 was considered statistically significant. *P* values were showed as: ns, not significant; *, *P*< 0.05; **,*P*< 0.01; ***, *P*< 0.001.

## Results

### Identification of prognostic cellular senescence-*related gene* of bladder cancer in the TCGA cohort

**Figure 1:**
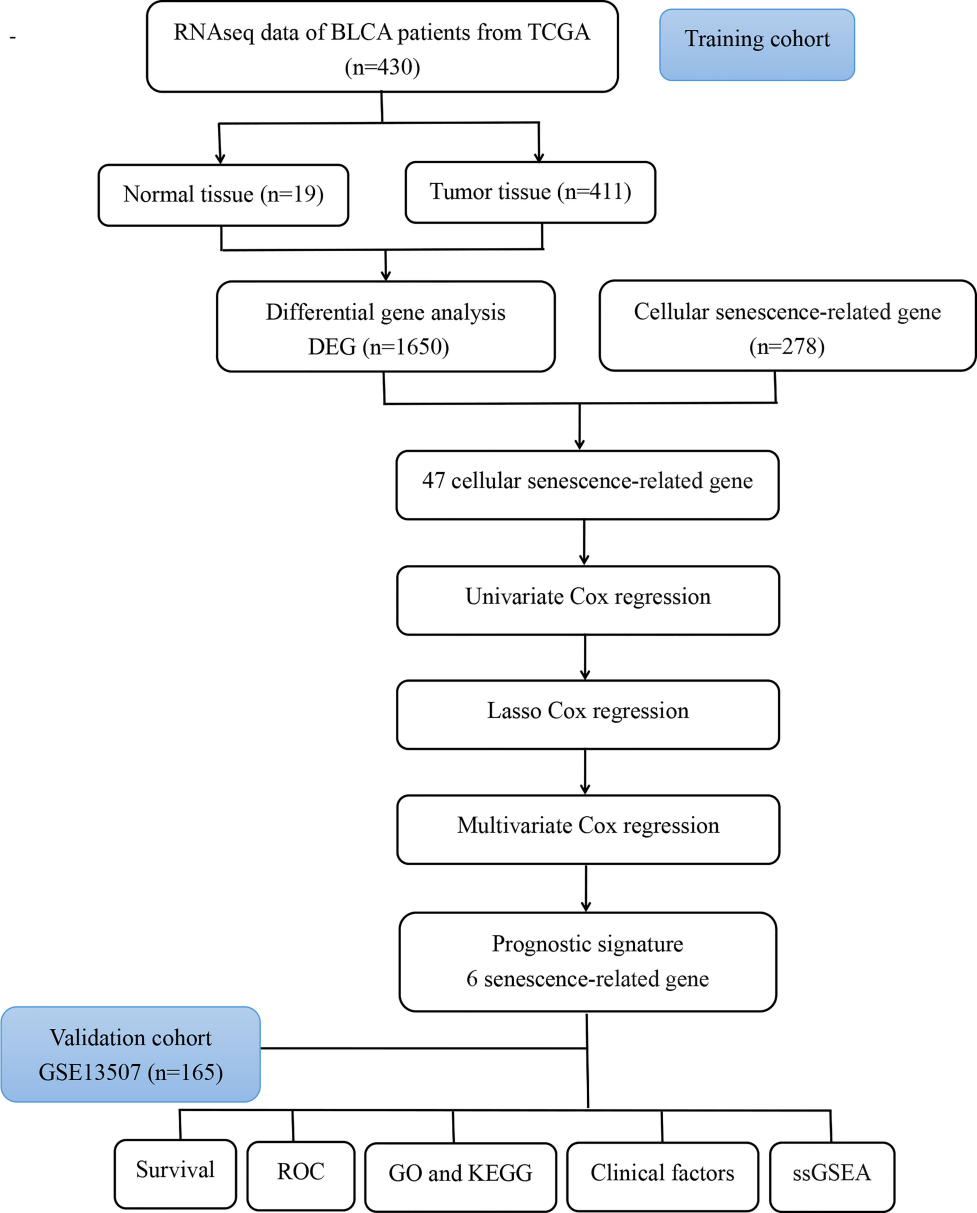
Flow chart of the analysis process in our study.

**Figure 2:**
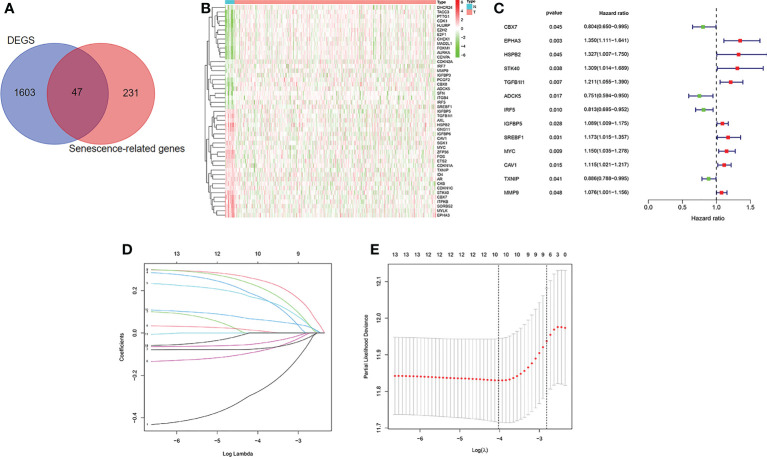
Identification of the candidate senescence-related genes in the TCGA cohort. **(A)**. Venn diagram to identify differentially expressed senescence-related genes between normal and tumor tissue. **(B)**. The 47 overlapping genes were differently expressed in normal and tumor tissue. **(C)**. Forest plots showing the results of the univariate Cox regression analysis between overlapping genes and overall survival. **(D, E)** LASSO Cox regression analysis was conducted to screen the key genes.

The flow diagram of the study is presented in **Figure 1**. Among 278 cellular senescence-related genes, 47 were differentially expressed between tumor samples and normal samples, including 24 downregulated genes and 23 upregulated genes (**Figures 2A, B**). Through univariate Cox regression analysis, we identified 13 prognostic cellular senescence-related genes associated with overall survival, as detailed in **Figure 2C**.

### Construction of a prognostic model in the training cohort

**Figure 3:**
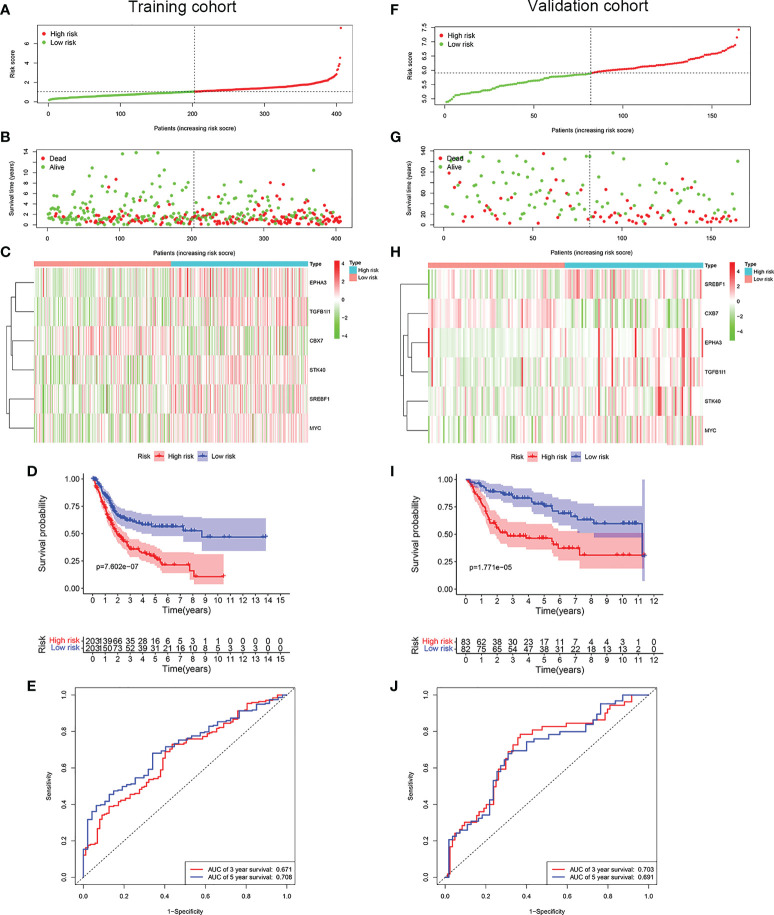
Construction of a prognostic model of senescence-related genes in the TCGA cohort and validation in GSE13507 cohort. **(A)** Distribuion of patients’ risk scores, **(B)** survival status, **(C)** Prognostic signature signal heatmap, **(D)** Kaplan-Meier curves for the overall survival, and **(E)**. ROC curve of the prognostic signature in the TCGA cohort. **(F)** Distribuion of patients’ risk scores, **(G)** survival status, **(H)** Prognostic signature signal heatmaps, **(I)**. Kaplan-Meier curves for the overall survival, and **(J)**. ROC curve of the prognostic signature in the GSE13507 cohort.

LASSO Cox regression analysis was conducted to screen the key genes among the cellular senescence-related genes based on univariate Cox regression analysis results (**Figures 2D, E**). Then, ten genes were selected into multivariate Cox regression analysis according to the optimal value of λ. Finally, six genes signature, namely, CBX7, EPHA3, STK40, TGFB1I1, SREBF1, and MYC, was constructed. The risk score of each patient was calculated according to the following formula: risk score = (-0.474 × the expression level of CBX7) + (0.354 ×the expression level of EPHA3) + (0.283 × the expression level of STK40) + (0.244 × the expression level of TGFB1I1) + (0.287× the expression level of SREBF1) + (0.104 × the expression level of MYC). Patients were stratified into high risk group (n=203) and low risk group (n=203) based on the median risk score. As shown in **Figures 3A, B**, patients in the high risk group had higher probability of death than that in the low risk group. Patients in the high risk group had high expression of EPHA3, TGFB1I1, STK40, SREBF1, and MYC but low expression of CBX7 (**Figure 3C**). The Kaplan-Meier analysis demonstrated that patients in the high risk group had significantly poorer overall survival (OS) than those in the low risk group (**Figure 3D**). The ROC curves was conducted to evaluate the predictive accuracy of the risk model for OS.The results demonstrated that the area under ROC curve (AUC) reached 0.671 at 3 year and 0.708 at 5 year (**Figure 3E**).

### Validation of the prognostic risk model in the validation cohort

To evaluate whether the risk model constructed from the TCGA cohort was robust, the patients from the GSE13507 were categorized into high risk group and low risk group according to the median value calculated with the same formula as the training cohort. Similar to the results obtained from the training cohort, patients in the high risk group of validation cohort were more likely to encounter a higher probability of death (**Figures 3F, G**). Likewise, patients in the high risk group had high expression of EPHA3, TGFB1I1, STK40, SREBF1, and MYC but low expression of CBX7 (**Figure 3H**). As expected, patients exhibited worse OS in the high risk group. (**Figure 3I**). The AUC value reached 0.703 at 3 year, 0.691 at 5 years (**Figure 3J**).

### Correlation between the cellular senescence-*related gene* signature model and the clinicopathological features

**Figure 4:**
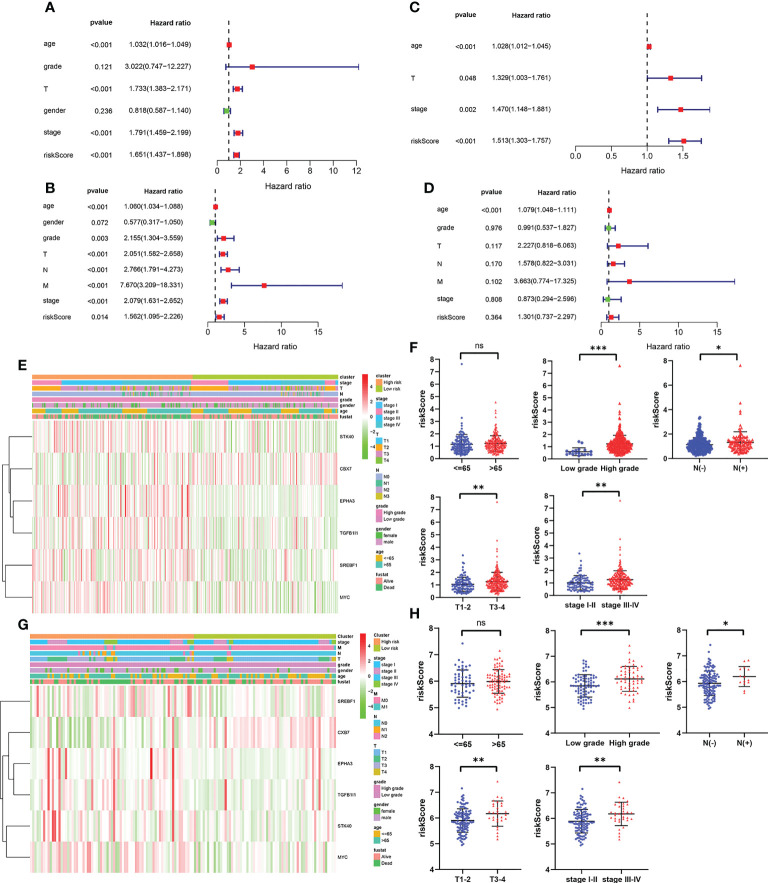
The independent prognostic factors for overall survival. **(A, B)** Univariate and multivariate cox regression of prognostic factors in the TCGA cohort. **(C, D)** Univariate and multivariate cox regression of prognostic factors in the GSE13507 cohort. **(E, F)** The relationship between risk score and clinicopathological parameters in the TCGA cohort. **(G, H)** The relationship between risk score and clinicopathological parameters in the GSE13507 cohort. *P* values were showed as: ns, not significant; *, *P* < 0.05; **,*P* < 0.01; ***, *P* < 0.001.

To evaluate whether the prognostic model based on cellular senescence-related gene was an independent risk factor for OS, the Cox regression analysis was performed. The results obtained from univariate Cox regression analysis indicated that the risk score was significantly correlated with OS in the training cohort (HR 1.651, 95% CI:1.437–1.898, *P* < 0.001) and validation cohort (HR 1.562, 95% CI: 1.095–2.226, *P* = 0.014) (**Figures 4A, C**). Multivariate Cox regression demonstrated that riskScore (HR 1.513, 95% CI 1.303–1.757, *P* < 0.001) would be an independent risk factors for OS in the training cohort (**Figure 4B**), however, the result was not observed in the validation cohort (**Figure 4D**).

Subsequently, the relevance between the risk score and clinicopathological parameters of patients was evaluated and exhibited as a heatmap in the training cohort (**Figure 4E**) and validation cohort (**Figures 4G**). The results confirmed that the risk score had strong correlations with the tumor grade, N stage, T stage and TNM stage in the training cohort (**Figure 4F**). The similar results were observed from the validation cohort (**Figure 4H**). All these results verified that as the risk score increased, the probability of progressing to a later clinical phenotypes gradually increased, suggesting that cellular senescence-related gene signature would be a valuable cancer prognostic model in the progression of BCa.

### Analysis expression of the genes from the signature in bladder cancer patients

We detected mRNA expression of the genes from this signature in 5 paired human BCa tissues and adjacent normal tissues. The results indicated that the expression of EPHA3, TGFB1I1, STK40 and CBX7 were down-regulated in BCa tissues than that in adjacent normal tissues. The expression of SREBF1 and MYC were up-regulated in BCa tissues than that in adjacent normal tissues (**Supplementary Figure 1**).

### Functional enrichment analyses in training cohort

**Figure 5:**
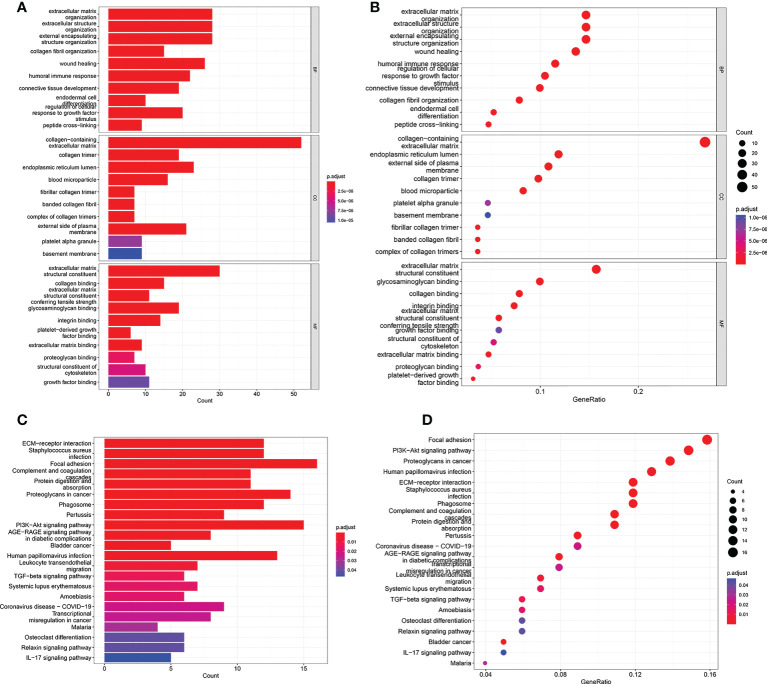
GO and KEGG analyses in TCGA cohort. **(A, B)** GO enrichment analysis in the TCGA cohort. **(C, D)** KEGG enrichment analysis in the TCGA cohort.

To investigate underlying biological functions and pathways that were related to the risk score, the GO enrichment and KEGG pathway analyses were performed using the DEGs between the high risk group and low risk group. The GO analysis results demonstrated that the biological process (BP) of DEGs mainly focused on extracellular matrix organization, extracellular structure organization, humoral immune response, regulation of cellular response to growth factor, and so on (**Figures 5A, B**). KEGG pathway analysis revealed that DEGs were significantly enriched in PI3K-Akt signaling pathway, Proteoglycans in cancer, ECM-receptor interaction, AGE-RAGE signaling pathway, Transcriptional misregulation in cancer, TGF-beta signaling pathway, Bladder cancer, IL-17 signaling pathway (**Figures 5C, D**).

### Differential immune infiltration characteristics landscape

**Figure 6:**
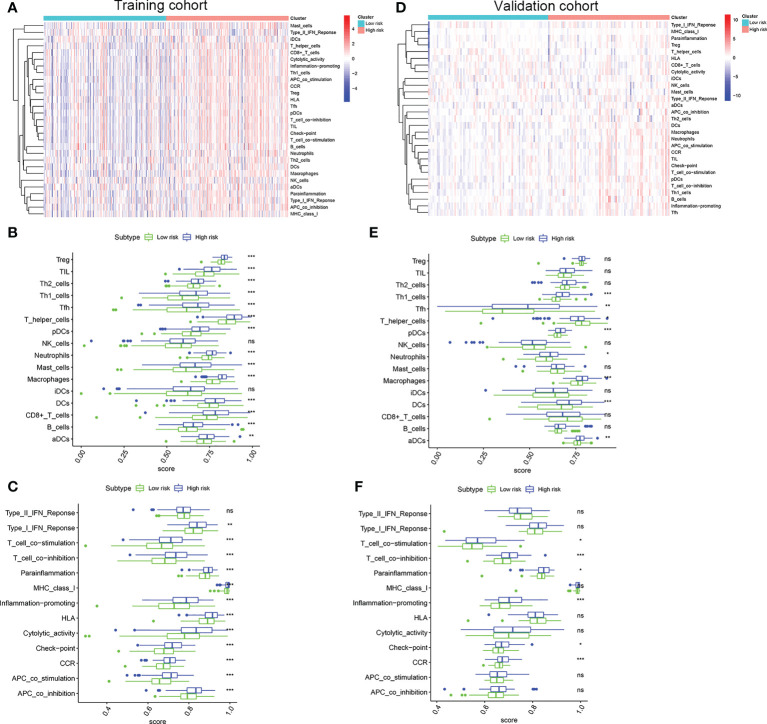
Comparison of the ssGSEA scores. **(A)** Relationship heatmap of the risk scores and ssGSEA scores, **(B)** Box plots showing the scores of immune cells, **(C)** Box plots showing the scores of immune function between the high risk and low risk group in the TCGA cohort. **(D)** Relationship heatmap of the risk scores and ssGSEA scores, **(E)** Box plots showing the scores of immune cells, **(F)** Box plots showing the scores of immune function between the high risk and low risk group in the GSE13507 cohort. *P* values were showed as: ns, not significant; *, *P* < 0.05; **,*P* < 0.01; ***, *P* < 0.001.

To evaluate the correlation between the risk score and immune activity, the ssGSEA algorithm was performed for the purpose of calculating the infiltrating scores of the different immune cell subsets, as well as immune-related function. The scores of Treg, TIL, Th2_cells, Th1_cell, Tfh, T_helper_cells, pDCs, Neutrophils, Mast_cells, Macrophages, iDCs, DCs, CD+_T_cells, B_cells and aDCs were significantly higher in high risk group than that in the low risk group in the training cohort (**Figures 6A, B**), suggesting immune cell abundance was significantly correlated with the riskScore. In addition, as shown in **Figure 6C**, the scores of immune function in the high risk group was stronger than that in the low risk group. Interestingly, the scores of Th1_cell, Tfh, T_helper_cells, pDCs, Neutrophils, Macrophages, DCs, and aDCs were significantly higher in the high risk group than that in the low risk group in validation cohort (**Figures 6D, E**). Moreover, compared with the low risk group, the scores of T_cell_co-stimulation, T_cell_co-inhibition, Inflammation-promoting, Check-point, CCR were significantly higher in the high risk group (**Figure 6F**). All these results, together, confirmed that the risk score had strong correlations with immune cell infiltration and immune-related function.

**Figure 7:**
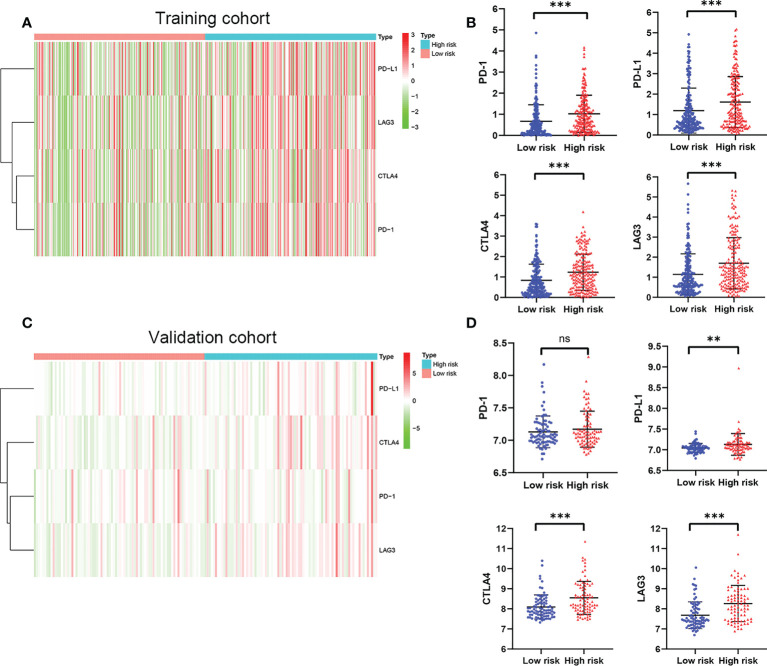
The expression of immune checkpoint molecules including PD-1, PD-L1, LAG3 and CTLA4. **(A)** Heatmap of immune checkpoint molecules expression, **(B)** Box plots showing the checkpoint molecules expression between the high risk and low risk group in the TCGA cohort. **(C)** Heatmap of immune checkpoint molecules expression, **(D)** Box plots showing the checkpoint molecules expression between the high risk and low risk group in the GSE13507 cohort. *P* values were showed as: ns, not significant; **,*P* < 0.01; ***, *P* < 0.001.

Considering the important role of immune checkpoint molecules such as PD-1, PD-L1, LAG3 and CTLA-4 in tumor immune microenvironment, the expression levels of these molecules were explored between two groups. Interestingly, PD-1, PD-L1, LAG3 and CTLA-4 of the high risk group were highly expressed in the training cohort (**Figures 7A, B**). In addition, we utilized validation cohort for exploring the molecules expression levels. Results presented that although the expression of PD-1 was no statistical difference, PD-L1, LAG3 and CTLA-4 were significantly high in the high risk group (**Figures 7C, D**).

### Differences in inflammatory response–related genes characteristics and EMT phenotype between high and low risk group

**Figure 8:**
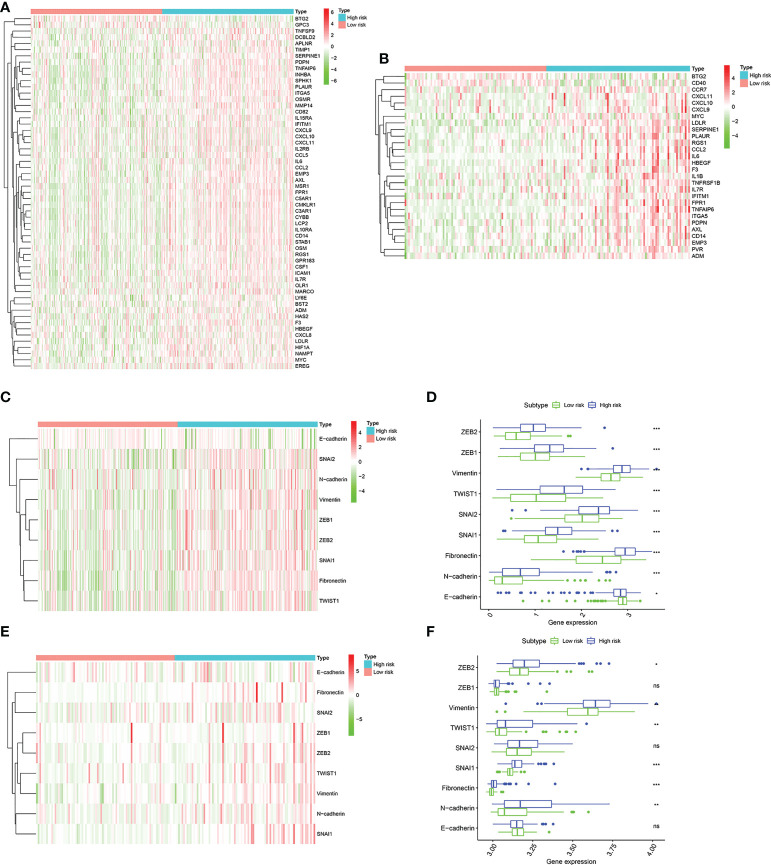
The landscape of inflammatory response–related genes and EMT hallmark genes including SNAI1, SNAI2, ZEB1, ZEB2, TWIST1, Vimentin, Fibronectin, N-cadherin, and E-cadherin. **(A)**. Heatmap of inflammatory response–related DEGs expression between the high risk and low risk group in the TCGA cohort. **(B)** Heatmap of inflammatory response–related DEGs expression between the high risk and low risk group in the GSE13507 cohort. **(C)** Heatmap of EMT hallmark genes expression, **(D)**. Box plots showing the EMT hallmark genes expression between the high risk and low risk group in the TCGA cohort. **(E)** Heatmap of EMT hallmark genes expression, **(F)**. Box plots showing the EMT hallmark genes expression between the high risk and low risk group in the GSE13507 cohort. *P* values were showed as: ns, not significant; *, *P* < 0.05; **,*P* < 0.01; ***, *P* < 0.001.

Due to the close connection between the senescence-associated secretory phenotype (SASP) and inflammatory response and epithelial mesenchymal transition (EMT), we decided to further investigate the differences in inflammatory response–related genes characteristics and EMT phenotype between two groups. First, The inflammatory response–related DEGs were identified between two groups by the “limma” R package based on the screening criteria of false discovery rate (FDR) < 0.05 and |log2FC| > 1. We found that these inflammatory response–related DEGs were highly expressed in high risk group and lowly expressed in low risk group in training cohort (**Figure 8A**). The similar results were observed from the validation cohort (**Figure 8B**). Next, EMT hallmark genes expression were explored between high and low risk group. As illustrated in **Figures 8C, D**, significantly higher expression of EMT hallmark genes including SNAI2, N-cadherin, Vimentin, ZEB1, ZEB2, SNAI1, Fibronectin and TWIST1 were observed in high risk group in the training cohort, along with the low expression of E-cadherin, suggesting that risk score was positively correlated with cell EMT pathway. Subsequently, these genes expression levels were explored in validation cohort. Results presented that although the expression of ZEB1, SNAI2 and E-cadherin were no statistical difference, ZEB2, TWIST1, SNAI1, Fibronectin and N-cadherin were significantly higher in the high risk group (**Figures 8E, F**), which was in accordance with our results that risk score was positively correlated with cell EMT pathway.

## Discussion

BCa is an extremely frequent genitourinary malignancy. Despite the therapeutic strategies and individualized therapies improvement, a significant proportion of patients with muscle invasive bladder carcinoma (MIBC) undergoing radical cystectomy still experience local recurrence and distant metastasis. Unfortunately, effective clinical treatments for these patients are relatively limited. Therefore, it is of great importance to identify novel molecular biomarkers to better advance cancer therapies and predict the prognostic of patients with BCa.

Cellular senescence is a state of durable growth arrest induced by various stresses (21). Several common features of cellular senescence include high expression of the cell cycle inhibitor p16^Ink4a^, and a distinctive senescence-associated secretory phenotype (SASP) that involves cytokines, chemokines, matrix metalloproteinases, growth factors and angiogenic factors (22). Emerging evidence has shown that cellular senescence plays a crucial role in tumor microenvironment (TME) and tumor growth (23, 24). In this study, the prognostic signature was constructed based on senescence-related genes in the TCGA cohort. Then, we validated the predictive power of the model in the GSE13507 cohort. The vast majority of patients in the TCGA are MIBC. The GSE13507 dataset include 101 NMIBC and 62 MIBC. Finally, six genes, including CBX7, EPHA3, STK40, TGFB1I1, SREBF1, and MYC were filtered out to construct the prognostic model.

CBX7 (Chromobox protein homolog 7) is reported to belong to the Chromobox protein family (25). Previous studies revealed that CBX7 expression was down-regulated in multiple human carcinomas and the loss of CBX7 expression was associated with increasing malignancy (26, 27). A recent study indicated that CBX7 acted as a tumor suppressor in BCa and could suppress cancer cell aggressiveness by inhibiting ERK signaling (25). EPHA3 is a member of the Eph receptor tyrosine kinases and can bind cell membrane ligands to mediate cell communication regulate biological function, including tumour growth, angiogenesis and metastasis  (28). EPHA3 is one of the potential anticancer targets, with up-regulation and tumor-promoting roles in a range of human cancers (29). However, the expression pattern and function of EPHA3 in BCa remains unclear, and further exploration are needed. STK40 (Serine/threonine kinase 40), also known as SHIK, which had been confirmed as a negative regulator of NF-κB transcription (30).

A recent evidence displayed that STK40 acted as a tumor inhibitor in patients with Triple-negative breast cancers (31). The expression details and functions of STK40 in various cancer types still warrants further investigation. TGFB1I1, a scaffold protein, is also known as HIC-5, which can be expressed under the induction of TGF-β and hydrogen peroxide (32). Previous studies revealed that TGFB1I1 acted as key roles in a variety of pathological processes, including liver fibrosis (32), atherosclerosis (33), tumorigenesis and progression (34). TGFB1I1 have been verified to be acted as an oncogene in several cancers, including esophageal squamous cell carcinoma (35), breast tumor (36), ovarian cancer (37), and osteosarcoma (38). SREBP1, a well-recognized transcriptional regulator of lipid metabolism (39). Previous studies have verified high expression of SREBP1 was related with a poor prognosis in multiple human tumors (40–42). MYC, one of the most frequently investigated proto-oncogene, has been reported as one of the most highly amplified oncogene contributes to the initiation and development of many human cancers (43, 44). A recent study revealed that MYC inhibitor not only suppressed tumor growth in mice, but also increased T cell immune infiltration, enhanced PD-L1 expression on tumors, and increased tumor sensitivity to anti-PD1 immunotherapy (45).

The patients were divided into high risk and low risk group based on the median risk score. Compared to the low risk group, patients in the high risk group had a higher probability of death. Survival analyses indicated that patients with high risk had a significantly poor prognosis. ROC curves indicated that higher consistency was existed between actual and predicted survival rate. Consistently, the percentage of patients with worse malignant phenotype, such as higher tumor grade, lymph node metastasis, and advanced TNM stage, was significantly higher in the high risk group.

We further reproduce the model in validation cohort to confirm the robustness of the risk model. As expected, results revealed that patients in the high risk group had a significantly increased risk of poor prognosis than that in the low risk group, which were consistent with our previous results. GO and KEGG analysis were performed based on DEGs between different risk groups to investigate the biological processes and signaling pathways that were related to the risk score. The results presented that DEGs were significantly enriched in extracellular matrix organization, humoral immune response, regulation of cellular response to growth factor, PI3K-Akt signaling pathway, TGF-beta signaling pathway, Bladder cancer, IL-17 signaling pathway. These biological processes are involved in immune biological processes and pathways (46–49).The extracellular matrix (ECM) is a combination of proteins and proteoglycans with structural and functional roles (50). Proteases and matrix metalloproteinases (MMPs) participate in ECM remodelling. Many evidence indicated that MMPs, such as MMP-7, MMP-10, associated with the progression in patients with BCa (51, 52). BCa is an immunogenic. Numerous studies found the influence of the immune microenvironment on BCa development and immunotherapy was applied for the treatment of BCa (53, 54). The development and progression of BCa has been associated with abnormal expression of a number of genes or aberrant activation of signaling pathways. Among them, PI3K-Akt pathway abnormally activated is crucial for BCa progression (55). Ruan et al. reported that inhibition of PI3K-Akt pathway significantly inhibited migration and invasion of BCa cells (56). TGF-β expression is up-regulated in tumor cells and TGF-β signaling pathway was notably associated with several hallmarks of cancer, such activating invasion and metastasis, inducing angiogenesis and drug resistance (57). Chen et al. found that TGF-β facilitated BCa cell proliferation, migration and invasion both *in vitro* and *in vivo* by inducing EMT (58). Liang et al. reported that ablation of TGF-β signaling suppressed the BCa cell proliferation and EMT, hence inhibited BCa progression in a BCa mouse model (59).The IL-17 cytokine family includes six ligands (IL-17A to IL-17 F) and five receptors (IL-17RA to IL-17RE) (60). Previous study indicated that IL-17F protein served as an oncogene in BCa (61). IL-17A levels were significantly elevated in peripheral blood in patients with BCa than that in healthy control (62). Wang et al. found that IL-17A facilitated BCa growth in animal experiments (63).

BCa is considered as an immune cell infiltrating tumor. At present, many immune checkpoint inhibitors, including atezolizumab, durvalumab, nivolumab, pembrolizumab, and avelumab, have been approved by FDA for the treatment of advanced urothelial carcinoma (64–70). In this study, two risk group confirmed by us had significantly different immune characteristics. High risk group had a significantly correlated with higher infiltrating scores of immune-cell and immunity-related pathways. Moreover, the expression of immune checkpoint molecules including PD-1, PD-L1, LAG3, and CTLA4 was significantly higher in the high risk group in training cohort. Similar to this results, in the validation cohort, the patients with high risk had a significantly higher expression of PD-L1, LAG3, and CTLA4. These results suggested that patients in the high risk group may have a more favorable response to immune checkpoint inhibitor therapy than that in the low risk group.

Inflammation were widely involved in the tumorigenesis and progression of tumor, immune escape, and tumor microenvironment formation (71, 72). We further investigated the inflammatory response–related genes (IRGs) expression abundance between the high risk and the low risk group. It is found that most of the differentially expressed IRGs were significantly higher expression in the high risk group than that in the low risk group. Moreover, based on the expression of EMT hallmark genes including SNAI2, N-cadherin, Vimentin, ZEB1, ZEB2, SNAI1, Fibronectin, TWIST1 and E-cadherin, we found that patients with high risk score distinctly exhibited a mesenchymal phenotype, suggesting a higher tumor malignancy.

In our study, six genes signature, namely, CBX7, EPHA3, STK40, TGFB1I1, SREBF1, and MYC, was constructed. Sun et al. identified a four-cell-senescence-regulator-gene prognostic index to predict the prognosis of patients with BCa (73). These four genes were PSMD14, PSMB5, PRPF19 and TPR. Zhou et al. developed an 8 immunosenescence-related gene pair signature to evaluate the overall survival of patients with BCa (74). These 8-gene pair were EGFR∣MAPK1, TFRC∣IRF1, ADIPOR2∣GBP2, CTSS∣THBS1, GBP2∣CCN2, PSMD11∣SRC, KIR2DL4∣NOX4, and MAP2K1∣ELAVL1. In TCGA cohort and GSE13507 cohort, we found that the high risk group and low risk group identified using cellular senescence-related gene in our study is not similar to the high risk group and low risk group identified in the Sun et al. and Zhou et al. papers (**Supplementary Tables 3** and **4**).

The risk score using gene signatures proposed in the Sun et al. and Zhou et al. papers were calculated. In TCGA cohort, the risk score calculated using gene signature proposed in our paper is not highly correlated with the risk score calculated using gene signatures proposed in the Sun et al. paper (r=0.282) and Zhou et al. papers (r=0.135). In GSE13507 cohort, the risk score calculated using gene signature proposed in our paper is not highly correlated with the risk score calculated using gene signatures proposed in the Sun et al. paper (r=0.115) and Zhou et al. paper (r=0.552) (**Supplementary Figure 2**). Therefore, the prognostic gene signature proposed in our study has uniqueness and novelty.

Some limitations should be acknowledged in our study. First, data from the BCa cohort in our clinical center are incomplete, thus, we cannot used our own BCa cohort to validate the predictive power of the model. We will improve the clinical data in the future to further validate the predictive power of the model. Second, as our prognostic model was constructed and validated with retrospective data, a multi-center prospective study with larger population is required to confirm the clinical value of the model. Third, further experimental studies are needed to clarify the biological regulatory mechanisms of cellular senescence-related genes in the progression of BCa.

## Conclusion

In summary, we established a novel prognostic model for BCa based on cellular senescence-related genes. Moreover, the model is capable of providing a reliable predictor for OS, clinical characteristics, and immune infiltration, which can serve as a valuable biomarker for bladder prognosis and progression.

## Data availability statement

The raw data supporting the conclusions of this article will be made available by the authors, without undue reservation.

## Ethics statement

The study was conducted in accordance with the Declaration of Helsinki, and the protocol was approved by the Ethics Committee of the First Affiliated Hospital of Nanchang University. The use of all samples was approved by the ethics committee, and informed consent was obtained from all enrolled patients.

## Author contributions

LL and WX were responsible for the study design and writing. BG was mainly responsible for data analysis, and FL and PX was mainly responsible for data collection. LL and WX was responsible for manuscript review and providing constructive comments. All authors read and approved the final manuscript. All authors contributed to the article and approved the submitted version.

## Acknowledgments

We thank for using TCGA and GEO database for free.

## Conflict of interest

The authors declare that the research was conducted in the absence of any commercial or financial relationships that could be construed as a potential conflict of interest.

## Publisher’s note

All claims expressed in this article are solely those of the authors and do not necessarily represent those of their affiliated organizations, or those of the publisher, the editors and the reviewers. Any product that may be evaluated in this article, or claim that may be made by its manufacturer, is not guaranteed or endorsed by the publisher.

## Supplementary material

Supplementary Figure 1 Analysis expression of the genes from the signature in BCa patients.

Supplementary Figure 2 Analysis correlation of risk score between different studies.
