# Supplementary figures and images for: A novel prognostic model based on cellular senescence-related gene signature for bladder cancer
Source: Front Oncol. 2022 Nov 23;12:937951. doi: 10.3389/fonc.2022.937951 (PMC9727082; doi:10.3389/fonc.2022.937951)

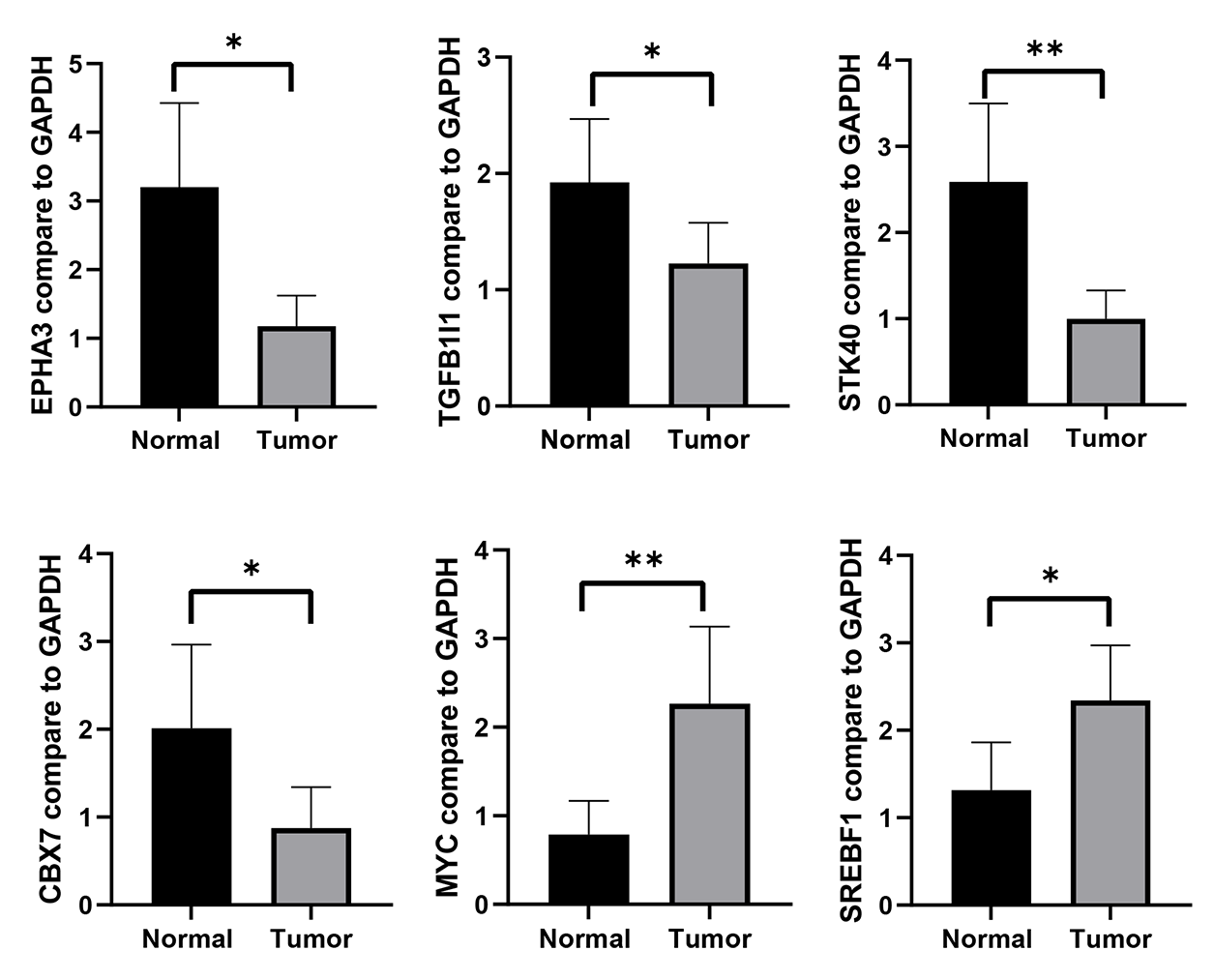

Supplement: Supplementary Figure 1 — Analysis expression of the genes from the signature in BCa patients. [file Image_1.tif]

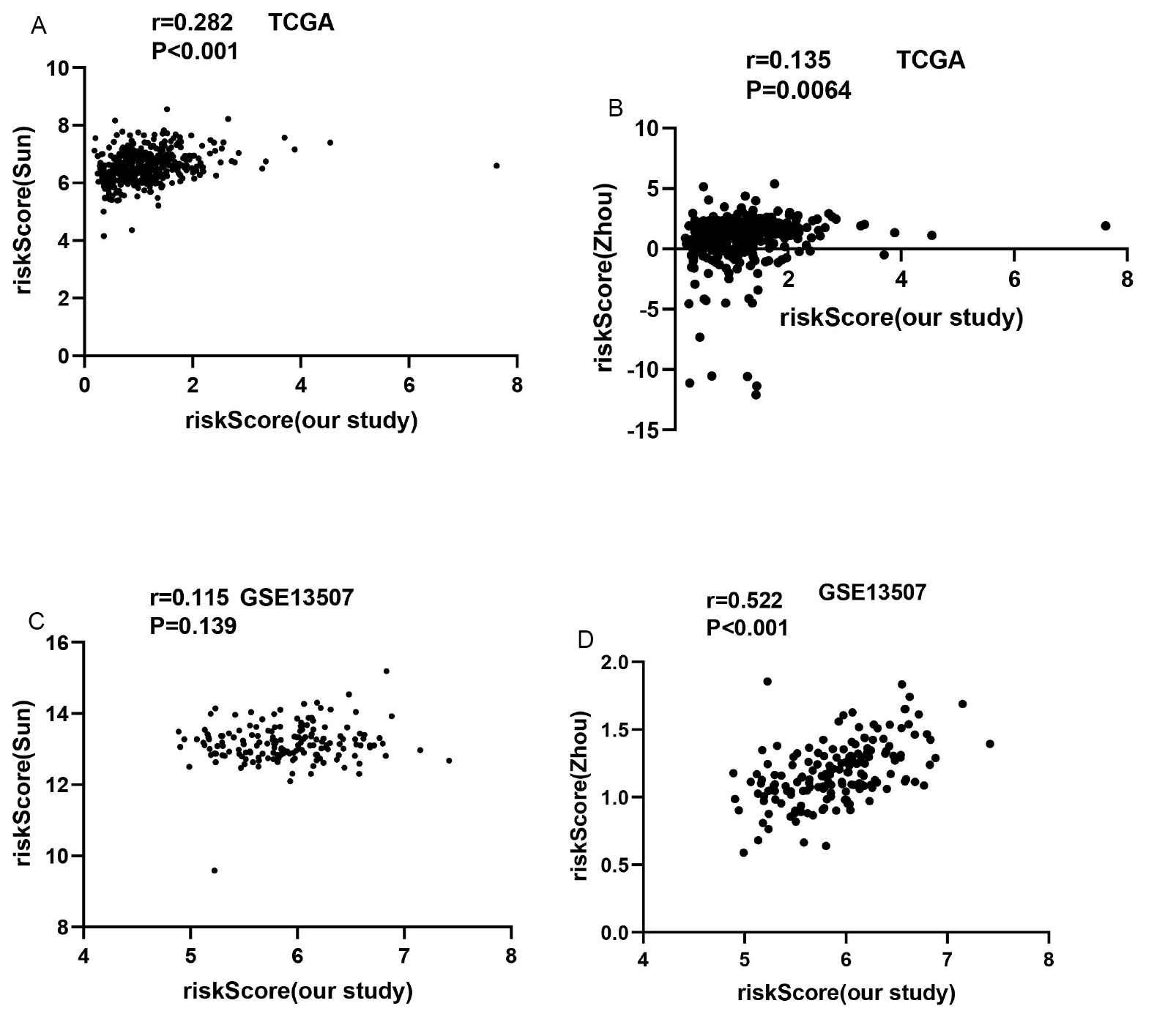

Supplement: Supplementary Figure 2 — Analysis correlation of risk score between different studies. [file Image_2.tif]
