# Supplementary material for: A novel prognostic model based on cellular senescence-related gene signature for bladder cancer
Source: Front Oncol. 2022 Nov 23;12:937951. doi: 10.3389/fonc.2022.937951 (PMC9727082; doi:10.3389/fonc.2022.937951)
Supplement: Supplementary file 4 [file Table_2.doc]

| Primer | Sequence |
| --- | --- |
| CBX7 Forward Primer | GCGTGCGGAAGGGTAAAGT |
| CBX7 Reverse Primer | GCTTGGGTTTCGGACCTCTC |
| EPHA3 Forward Primer | CTGCTCTGTTCTCGACAGCTT |
| EPHA3 Reverse Primer | CAGCTCCCCTTGAATTGTTTTTG |
| STK40 Forward Primer | ATGAAGCGGAGAGCATCAGAC |
| STK40 Reverse Primer | CCTCGCCAAACACTGCACTAT |
| TGFB1I1 Forward Primer | TACAGCACGGTATGCAAGCC |
| TGFB1I1 Reverse Primer | GCAACCGATCTAGCTCACAGAG |
| SREBF1 Forward Primer | ACAGTGACTTCCCTGGCCTAT |
| SREBF1 Reverse Primer | GCATGGACGGGTACATCTTCAA |
| MYC Forward Primer | GGCTCCTGGCAAAAGGTCA |
| MYC Reverse Primer | CTGCGTAGTTGTGCTGATGT |
| GAPDH Forward Primer | GGAGCGAGATCCCTCCAAAAT |
| GAPDH Reverse Primer | GGCTGTTGTCATACTTCTCATGG |

**Supplementary Table 2**.The sequence of primers in our study.
